# Variation in use of surveillance colonoscopy among colorectal cancer survivors in the United States

**DOI:** 10.1186/1472-6963-10-256

**Published:** 2010-09-01

**Authors:** Talya Salz, Morris Weinberger, John Z Ayanian, Noel T Brewer, Craig C Earle, Jennifer Elston Lafata, Deborah A Fisher, Bryan J Weiner, Robert S Sandler

**Affiliations:** 1 Health Outcomes Research Group, Department of Epidemiology and Biostatistics, Memorial Sloan-Kettering Cancer Center, 307 E. 63rd St., New York, NY 10065, USA; 2 Department of Health Policy and Management, Gillings School of Global Public Health, University of North Carolina at Chapel Hill, 1101d McGavran-Greenberg Hall, CB# 7411, Chapel Hill, NC 27599-7411, USA; 3 Division of General Medicine, Brigham and Women's Hospital; Department of Health Care Policy, Harvard Medical School, Department of Health Care Policy, Harvard Medical School, 180 Longwood Ave., Boston, MA 02115, USA; 4 Department of Health Behavior and Health Education, Gillings School of Global Public Health, University of North Carolina, 364 Rosenau Hall CB7440, Chapel Hill, NC 27599, USA; 5 Health Services Research Program, Cancer Care Ontario and the Ontario Institute for Cancer Research, Institute for Clinical Evaluative Sciences, Sunnybrook Health Sciences Centre, 2075 Bayview Avenue, Room G-106 Toronto ON, M4N 3M5, Canada; 6 Center for Health Services Research, Henry Ford Health System, 1 Ford Place, 3A, Detroit, MI 48202, USA; 7 Durham VAMC, HSR&D Center of Excellence, Duke University Medical Center, Department of Medicine, 508 Fulton Street, Building #6, Durham NC 27705, USA; 8 Department of Health Policy and Management, Gillings School of Global Public Health, University of North Carolina at Chapel Hill, 1101d McGavran-Greenberg Hall, CB# 7411, Chapel Hill, NC 27599-7411, USA; 9 Division of Gastroenterology and Hepatology, CB# 7555, 4157 Bioinformatics Building, University of North Carolina, Chapel Hill, NC 27599-7555, USA

## Abstract

**Background:**

Clinical practice guidelines recommend colonoscopies at regular intervals for colorectal cancer (CRC) survivors. Using data from a large, multi-regional, population-based cohort, we describe the rate of surveillance colonoscopy and its association with geographic, sociodemographic, clinical, and health services characteristics.

**Methods:**

We studied CRC survivors enrolled in the Cancer Care Outcomes Research and Surveillance (CanCORS) study. Eligible survivors were diagnosed between 2003 and 2005, had curative surgery for CRC, and were alive without recurrences 14 months after surgery with curative intent. Data came from patient interviews and medical record abstraction. We used a multivariate logit model to identify predictors of colonoscopy use.

**Results:**

Despite guidelines recommending surveillance, only 49% of the 1423 eligible survivors received a colonoscopy within 14 months after surgery. We observed large regional differences (38% to 57%) across regions. Survivors who received screening colonoscopy were more likely to: have colon cancer than rectal cancer (OR = 1.41, 95% CI: 1.05-1.90); have visited a primary care physician (OR = 1.44, 95% CI: 1.14-1.82); and received adjuvant chemotherapy (OR = 1.75, 95% CI: 1.27-2.41). Compared to survivors with no comorbidities, survivors with moderate or severe comorbidities were less likely to receive surveillance colonoscopy (OR = 0.69, 95% CI: 0.49-0.98 and OR = 0.44, 95% CI: 0.29-0.66, respectively).

**Conclusions:**

Despite guidelines, more than half of CRC survivors did not receive surveillance colonoscopy within 14 months of surgery, with substantial variation by site of care. The association of primary care visits and adjuvant chemotherapy use suggests that access to care following surgery affects cancer surveillance.

## Background

Colorectal cancer (CRC) survivors need ongoing preventive care even after their cancer treatment is complete. Because CRC survivors are at risk for both local recurrences and second primary cancers[1], clinical practice guidelines from several gastroenterological and oncological societies have long recommended routine surveillance colonoscopy for survivors who have been treated for cure[2-11]. By detecting second colorectal cancers and local recurrences early enough to treat, surveillance colonoscopy may increase treatment options and decrease mortality. Until recently, multiple guidelines offered conflicting recommendations for the timing of, and interval between, surveillance colonoscopies[2-10]. (Table 1) In 2006, the American Cancer Society and U.S. Multi-Society Task Force on Colorectal Cancer recommended ongoing colorectal surveillance for people with CRC via colonoscopy at 1 year after surgery, again at 3 years and, if normal, every 5 years thereafter[11].

**Table 1:** Summary of guidelines for first routine surveillance colonoscopy among colorectal cancer survivors, 1999-2006.

Year	Recommending agency	Timing of first colon exam
1999	American Society of Colon and Rectal Surgeons [3]	Colonoscopy or barium enema 1-3 years post-surgery
2000	American Society of Clinical Oncology [2]	Colonoscopy in 3-5 years
2003	American Cancer Society [4]	Colonoscopy within 1 year post-surgery
2003	U.S. Multisociety Task Force on Colorectal Cancer Screening and Surveillance [5]	Colonoscopy at 3 years post-surgery
2003	National Comprehensive Cancer Network [6]	Colonoscopy within 3 years
2004	American Society of Colon and Rectal Surgeons [7]	Colonoscopy in 1-3 years
2004	National Comprehensive Cancer Network [25]	Colonoscopy within 3 years
2006	National Comprehensive Cancer Network [10]	Colonoscopy in 1 year
2006	American Society for Gastrointestinal Endoscopy [32]	Colonoscopy at 1 year post-surgery
2006	U.S. Multisociety Task Force on Colorectal Cancer Screening and Surveillance [11]	Colonoscopy at 1 year post-surgery

Note: Table does not include recommendations for preoperative colonoscopies or those occurring postoperatively if preoperative colonoscopies are not possible.

Unfortunately, many survivors do not receive their first surveillance colonoscopy within 1 year after surgery, and some never undergo surveillance. Estimates of CRC survivors' receipt of colon exams (including colonoscopies) range from 52-61% within 18 months of diagnosis [12-16], 60-76% within 3 years[12,16-18], and only 51-80% within 5 years[12,14,17,19]. Of more concern, however, are differences in routine surveillance by survivor characteristics. Studies have suggested that factors such as race and ethnicity, age, marital status, income, geographic region, site of the tumor, comorbidities, and stage may be associated with whether survivors undergo appropriate surveillance[12-21].

Prior studies, however, have three important limitations. First, they used data from 1986 through 2004 [12-21], a time period that does not reflect newer treatment options. Second, these studies have included only patients from restricted age and insurance groups (e.g., Medicare recipients), clinic-based populations, or regional health systems[12-18,21]. Finally, previous studies have been limited to administrative data [12,13,16,17,20,21], which may not capture important details about survivors' clinical characteristics.

We used interview and medical record data from a recent nationwide population-based sample of CRC survivors to examine surveillance colonoscopy use. Specifically, we sought to: (1) estimate the rate of surveillance colonoscopy use among CRC survivors 14 months after surgery and (2) identify characteristics of survivors who received colonoscopy in that time period.

## Methods

### Study sample

We studied participants in the Cancer Care Outcomes Research and Surveillance (CanCORS) Consortium, a multi-regional U.S. population-based cohort study of lung and colorectal cancer. Detailed descriptions of CanCORS have been published elsewhere[22,23]. Briefly, patients diagnosed with CRC or lung cancer were identified an average of 1.8 months after diagnosis from 4 geographic regions (Northern California, Los Angeles, Alabama, and North Carolina); 15 Veterans Affairs (VA) Medical Centers; and 5 members of the Cancer Research Network, a network of managed care organizations designated by the National Cancer Institute to conduct research on cancer prevention and control[24]. Because colonoscopy records in the Northern California site and one of the managed care organizations were incomplete, those data were not included in this analysis (N = 944). CRC patients in CanCORS were diagnosed from May 2003 through October 2005. The research was approved by the Institutional Review Boards at each site and all subjects gave written informed consent.

### Inclusion and exclusion criteria

We restricted analyses to survivors who: (1) had stage I, II, or III CRC; (2) received surgery within one month of diagnosis (so that they had at least 14 months of follow-up time), (3) were alive 14 months after surgery (so that they had the opportunity to receive colonoscopy); and (4) had adequate medical record data. Participants who had local recurrences within the study period were excluded, unless the recurrence was preceded (and therefore possibly diagnosed) by a colonoscopy. Participants who had colonoscopies performed within 60 days after surgery were excluded because it is likely that this was due to complications form the initial surgery, was prompted by symptoms, or was conducted in place of a preoperative colonoscopy.

### Data sources

Data for this study came from two sources. First, for eligible participants providing informed consent, staff trained by a central CanCORS team conducted medical record audits, with quality control mechanisms in place. Data on cancer-related medical visits and procedures were extracted beginning 3 months before diagnosis through 15 months after diagnosis. Second, interviews with participants or their proxies (in the case of survivor illness) were conducted approximately 4 months after diagnosis. Interviews elicited information about symptoms, satisfaction with care, and other topics for which survivors are an important source of information. A centralized statistical and computing core derived key variables, including staging, comorbidity, and adjuvant treatment variables, from both abstracted medical records and participant interviews.

### Colonoscopy use

Our primary outcome was receipt of the first surveillance colonoscopy within 14 months of surgery. While some guidelines conflicted during the study period for the timing of the first routine surveillance (i.e., 1 versus 3 years after surgery or diagnosis), we chose the 14-month period, because 6 guidelines recommended a colonoscopy at 1 year [4,10,11] or said it would be appropriate[3,6,25]. In addition, the 14-month period allowed us to balance the time allowed for survivors to have surgery with adequate post-surgical follow-up analysis time within the available 15 months of post-diagnosis data.

### Covariates

Sociodemographic data included age (from medical records) and sex, race and ethnicity, income, insurance, marital status, and education (from interviews). Clinical data (predominantly from medical records) included site of the tumor, stage at diagnosis, presence and severity of comorbidities, cancer treatments, and visits to primary care physicians and medical oncologists. Staging was determined using collaborative staging, or if it was unavailable, from the best available data from the registry, medical records, or interviews. Comorbidity was assigned as none, mild, moderate, or severe using the Adult Comorbidity Evaluation (ACE-27) index[26-28]. Adjuvant treatment was defined as having had chemotherapy or radiation therapy within 6 months after surgery.

### Data analyses

Missing data were imputed using an iterative multivariable regression technique in Stata[29]. Analyses excluded dichotomous predictor variables that had less than 5% of the sample in one category. We used logistic regression to examine bivariate and multivariate relationships between survivor characteristics (clinical and demographic) and receipt of colonoscopy, using robust standard errors corrected for heteroskedasticity. We calculated odds ratios for the effect of each predictor on colonoscopy use and conducted Wald χ^2 ^tests of joint significance for categorical variables[30].

To describe the magnitude of the effect of dichotomous predictors on the likelihood of colonoscopy use, we computed average changes in predicted probability using a multi-step process. For each variable of interest, we subtracted the expected value of the dependent value when the independent variable was equal to 0 from the expected value of the dependent value when the independent variable was equal to 1. The difference is the average change in predicted probability resulting from having the population change from the first (reference) value of the predictor to the second (nonreference) value. See Madden et al. for an example of this methodology[31].

## Results

**Figure 1:**
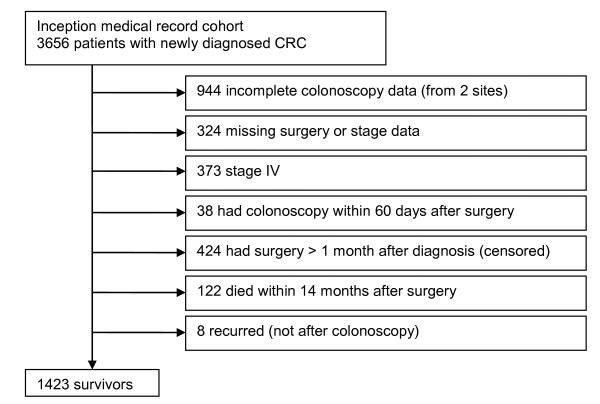
Flow diagram of study participants

Of 3,656 patients with newly-diagnosed CRC, 1,423 (39%) met all eligibility criteria and contributed data to analyses (Figure 1). Thirty-five participants (2%) did not complete interviews, and their interview data were imputed. Most survivors (79%) had a primary colon, as opposed to rectal, cancer. Demographic and clinical characteristics of the cohort appear in Table 2. Because of small cell sizes, we collapsed Asian, American Indian, Pacific Islander, mixed race, other race, and unknown race into a single category. Insurance status (i.e., whether participants had insurance), having had adjuvant radiation, and speaking English were excluded from regression analyses because of minimal variation in these variables.

**Table 2:** Characteristics of study sample (N = 1423)

	N	%
Age		
Less than 65 years	621	44
65 years and older	802	56
Annual income		
<$50,000	592	42
≥ $50,000	460	32
Site of disease		
Colon	1122	79
Rectum	259	18
Stage		
I	413	29
II	499	35
III	505	35
Comorbidities		
None	356	25
Mild	638	45
Moderate	256	18
Severe	173	12
Sex		
Male	833	56
Female	590	41
Marital status		
Married	792	56
Not married	580	41
Education		
Less than high school	682	48
High school or more	687	48
Race/ethnicity		
Non-Hispanic White	957	67
Non-Hispanic Black	257	18
Hispanic	84	6
Other	123	9
Site of care		
Alabama	306	22
Los Angeles	300	21
North Carolina	416	29
VA hospitals	166	12
Managed care	235	17
Insurance		
Insured	1232	87
Not insured	25	2
Speaks English		
Yes	1386	97
No	29	2
Saw a primary care provider		
Yes	868	61
No	553	39
Saw a medical oncologist		
Yes	1032	73
No	391	27
Had adjuvant chemotherapy		
Yes	590	41
No	833	59

Note: Percentages may not add to 100 because of missing data.

Only 49% of patients across all sites had a colonoscopy within 14 months after surgery. In bivariate analyses, colonoscopy use varied by severity of comorbidities, age, income, site of care, seeing a medical oncologist, and having adjuvant chemotherapy. (Table 3) In multivariate analysis (Table 3), the likelihood of receiving surveillance colonoscopy was greater among colon cancer than rectal cancer survivors (OR = 1.41, 95% CI: 1.05-1.90). Compared to survivors with no comorbidities, survivors with moderate or severe comorbidities were less likely to receive surveillance colonoscopy (OR = 0.69, 95% CI: 0.49-0.98 and OR = 0.44, 95% CI: 0.29-0.66, respectively). Those diagnosed at stage III were less likely to receive surveillance colonoscopy than those diagnosed at stage I (OR = 0.68, 95% CI: 0.47-0.98), although the stage variables were not jointly significant. Having seen a primary care provider in the first year after diagnosis (OR = 1.44, 95% CI 1.14-1.82) and having had adjuvant chemotherapy (OR = 1.75, 95% CI: 1.27-2.41) were both positively associated with colonoscopy use.

**Table 3:** Relationships between patient characteristics and colonoscopy use within 14 months of surgery (N = 1423)

	Had colonoscopy N (%)	Bivariate		Multivariate	
		OR	p	OR	p
All	692 (49)				
Age					
<65 years	328 (53)	1.35	<0.01	1.05	0.69
≥65 years	364 (45)	1.00	-	1.00	-
Sex					
Male	393 (47)	0.87	0.19	0.87	0.26
Female	299 (51)	1.00	-	1.00	-
Site of tumor					
Colon	576 (50)	1.28	0.08	1.41	0.02
Rectum	116 (44)	1.00	-	1.00	-
Stage					
I	192 (46)	1.00	-	1.00	-
II	244 (49)	1.01	0.92	0.89	0.44
III	255 (50)	1.12	0.30	0.68	0.04
Comorbidities					
None	197 (55)	1.00	-	1.00	-
Mild	330 (52)	1.25	0.04	0.92	0.56
Moderate	110 (43)	0.76	0.046	0.69	0.04
Severe	55 (32)	0.45	<0.01	0.44	<0.01
Race/ethnicity					
Non-Hispanic White	468 (49)	1.00	-	1.00	-
Non-Hispanic Black	120 (47)	0.91	0.48	0.89	0.48
Hispanic	38 (45)	0.87	0.54	0.95	0.83
Other	66 (54)	1.25	0.24	1.50	0.05
Education					
<high school	328 (46)	1.00	-	1.00	-
≥High school	364 (51)	1.22	0.07	1.07	0.62
Annual income					
<$50,000	364 (44)	0.66	<0.01	0.74	0.05
≥$50,000	328 (55)	1.00	-	1.00	-
Marital status					
Married	415 (51)	1.20	0.09	1.03	0.79
Not married	277 (46)	1.00	-	1.00	-
Site of care					
Alabama	156 (51)	1.13	0.35	1.92	<0.01
Los Angeles	144 (48)	0.97	0.81	1.49	0.03
North Carolina	236 (57)	1.58	0.00	2.32	<0.01
VA hospitals	66 (40)	0.67	0.02	1.38	0.16
Managed care	90 (38)	1.00	-	1.00	-
Saw a primary care provider					
Yes	440 (51)	1.22	0.06	1.44	<0.01
No	252 (46)	1.00	-	1.00	-
Saw a medical oncologist					
Yes	530 (51)	1.49	<0.01	1.23	0.17
No	162 (41)	1.00	-	1.00	-
Had adjuvant chemotherapy					
Yes	332 (56)	1.69	<0.01	1.75	<0.01
No	360 (43)	1.00	-	1.00	-

Note: OR = odds ratio.

Sites of care were strongly associated with colonoscopy use. Compared to survivors enrolled through managed care organizations in the Cancer Research Network, survivors in North Carolina were more likely to receive colonoscopy (OR = 2.32, 95% CI: 1.65-3.27), as were survivors in Los Angeles (OR = 1.49, 95% CI: 1.03-2.15) and Alabama (OR = 1.92, 95% CI: 1.31-2.81). There was no significant difference between rates of surveillance colonoscopy in the VA sites and the managed care organizations.

The magnitude of the statistically significant dichotomous predictors was calculated as an average marginal effect, indicating the change in probability of receiving a colonoscopy if every study participant first had the reference value of an independent variable and then changed to the indicator value. Assuming the relationship between these factors and colonoscopy use is causal, if every participant in the study changed from not seeing a primary care provider to seeing a primary care provider, the likelihood of receiving a colonoscopy would increase by 8 percentage points on average. Similarly, a change from not having adjuvant chemotherapy to having adjuvant chemotherapy would increase the likelihood of receiving a colonoscopy by 13 percentage points, on average.

## Discussion

Guidelines recommend that CRC survivors receive regular surveillance via colonoscopy[2-7,10,11,25,32]. Previous studies found that the rate of surveillance colonoscopy among CRC survivors is low; however, these studies used older data sets which limit generalizability[12-15,17,19,21,33]. Using rich medical record and interview data from a recent multi-regional population-based cohort of CRC survivors, we extended prior research on surveillance colonoscopy among CRC survivors. This study using medical record abstraction included survivors with a range of insurance types, ages, and ethnic backgrounds, strengthening inferences about external validity based on study findings.

We found that only 49% of CRC survivors received surveillance colonoscopy. This rate is somewhat lower than previously-reported rates at 18 months (52-61%)[12-15]. The prior studies' slightly higher estimates may simply be due to additional analysis time or inclusion of other colon examinations (such as sigmoidoscopy and barium enema)[12-15]. However, given that colonoscopies were 70-97% [12-14] of all colon examinations, the use of other examinations does not fully account for the higher utilization rate in earlier studies. Cooper and Payes[16] suggested that survivors may be delaying their first colonoscopy until after 1 year. It is possible that since 2002, the average time of receipt of the first postoperative colonoscopy has become further delayed beyond 14 months. Notably, sensitivity analyses suggested that are findings are robust with respect to length of post-surgical follow-up duration (13 months, 13.5 months, 14.5 months, and greater than 15 months after diagnosis). Moreover, excluding patients with both rectal and colon cancer (versus only one of the two) yielded identical results for both the direction and statistical significance of effect sizes.

There is large variation in surveillance colonoscopy among subpopulations. The greatest variation was found between study sites, where we found rates ranging from 37% to 57%. This finding is similar to the geographic variation reported using Surveillance Epidemiology and End Results (SEER) data and in a Canadian registry study[18,19,21,33]. This regional variation may be a function of differences in practice patterns or health care delivery systems. Our results show that the utilization of surveillance colonoscopy is lowest in managed care organizations. Because the sites of care are either geography-based, as in the Los Angeles and Alabama sites, or based around a health care system, as in the VA and the managed care sites, it is difficult to disentangle these different possible influences on colonoscopy use in the present study.

Clinical factors were strongly associated with colonoscopy use. Rectal cancer survivors were less likely to than those with colon cancer to receive colonoscopy, which is consistent with previous reports[14,16,17,20]. Because colonoscopies comprised 96-99% of all procedures performed, the higher use of sigmoidoscopy among rectal patients is unlikely to account for this finding[12,14,17]. Instead, less frequent colonoscopy use among rectal cancer survivors may reflect a selection bias. Rectal cancer survivors are more likely than colon cancer survivors to get neoadjuvant therapy (chemotherapy or radiation), but our criteria of surgery within one month excluded many survivors who received neoadjuvant therapy. Indeed, due to censoring, the proportion of participants who received neoadjuvant therapy fell from 9% to 1% among colon cancer survivors and from 30% to 0% among rectal cancer survivors. Because patients who receive neoadjuvant therapy may be more likely to have a timely colonoscopy, it is also possible that those who were most likely to adhere to colonoscopy guidelines were excluded from the analysis.

Survivors with more severe comorbidities were less likely to receive a colonoscopy. Previous studies using SEER data among Medicare fee-for-service populations have shown similar findings[16,18,20], although two studies[14,15] of managed care populations showed no effect of comorbidities on colonoscopy use. Patients with more severe comorbidities may have shorter life expectancies, leading physicians to be less likely to offer, and patients less likely to accept, surveillance colonoscopy. As well, survivors with more severe comorbidities may be too frail to undergo colonoscopies.

Seeing a primary care physician in the first year after diagnosis is associated with a higher likelihood of receiving a colonoscopy. Similarly, those individuals who received adjuvant chemotherapy were more likely to receive a colonoscopy. Two recent studies found that CRC survivors who were seen by both an oncologist and a primary care physician received the highest proportion of recommended ongoing cancer preventive care (such as cervical cancer screening and mammography) compared to survivors who saw either type of physician (or had seen neither)[34,35]. Primary care visits and chemotherapy use may be indicators of access to high-quality care and having a medical home, thereby increasing referral for timely surveillance colonoscopies. If primary care providers are responsible for most referrals, improving coordination of care as patients move from acute cancer care to ongoing care may increase adherence to colonoscopy guidelines. The Institute of Medicine has prioritized facilitating this transition, with the goal of improving ongoing care for cancer survivors[36].

Alternatively, prevention-oriented survivors may be more likely to visit a primary care physician, to seek out adjuvant chemotherapy to reduce the risk of recurrence, and, similarly, to request a colonoscopy to reduce their risk of cancer. Future longitudinal studies should address survivors' attitudes toward colonoscopy and CRC prevention and whether these attitudes affect colonoscopy use.

Sociodemographic factors were not related to colonoscopy use. Although previous studies have found associations between sociodemographic factors and colorectal surveillance, results have conflicted across studies[12-18,20,21]. Taken together with the current study, this suggests that sociodemographic factors are inconsistent predictors of colonoscopy use. Instead, the pattern of results appears to be that survivors are more likely to undergo surveillance colonoscopy if they have the best prognosis (those with mildest or no comorbidities, earlier stage, and use of adjuvant chemotherapy) or have a primary care physician.

This study has limitations. First, inclusion criteria of surgery within 1 month after diagnosis could limit the findings. However, sensitivity analyses that permitted longer lags between diagnosis and surgery, as well as a sensitivity analysis that did not limit timing of the surgery, showed similar results. It is still possible that those with later surgeries and those with early post-surgical colonoscopies had different patterns of colonoscopy use than their counterparts. Second, this study sample was largely insured, albeit to different extents and with different insurance types. Despite high levels of insurance, however, differences in surveillance colonoscopy use were observed in this study. Third, we were unable to assess the clinical impact of nonadherence in our sample. Fourth, as we could not assess reasons for colonoscopy use (i.e., diagnostic versus surveillance), some colonoscopies may have been diagnostic instead of for routine surveillance. This is unlikely, though, because Cooper et al[37] looked at indications for colonoscopy use among cancer survivors and found that 95% of colonoscopies performed were routine. Fifth, the study assessed colonoscopy use by 14 months after surgery. Although significant variations in colonoscopy use were apparent even in this limited time frame, a longer analysis period may have accommodated delays in scheduling colonoscopies and revealed additional colonoscopies that would still be adherent to clinical practice guidelines in effect during the study period. A longer analysis period also would have included colonoscopies that were delayed due to long wait times. Finally, survivors who were missing data on surgery and staging were excluded from the analytic sample, and this may have created a selection bias. Although we do not know whether those with missing surgery data did not have colorectal cancer surgery or whether their surgery data are missing, the colonoscopy surveillance guidelines are targeted only to patients who do have surgery. A subset of patients lacking surgery data presumably did not have surgery and are appropriately excluded from the analytic sample, limiting the bias in our estimates. Patients who lacked staging information had a 41% colonoscopy rate overall. The lower rate of colonoscopies compared to the rate in the analytic sample (49%) may simply reflect the presence of patients with advanced disease in the unstaged sample; patients with advanced disease are not appropriate candidates for surveillance colonoscopy.

## Conclusions

Despite guidelines, more than half of CRC survivors did not receive surveillance colonoscopy within 14 months of surgery, with substantial variation by site of care. The association of primary care visits and adjuvant chemotherapy use suggests that access to care following surgery affects cancer surveillance. Future studies should examine more closely the referral process for surveillance colonoscopies in order to identify clinically inappropriate variation across subgroups of survivors. Further clarifying which types of providers CRC cancer survivors routinely see for follow up care, when these visits occur, whether these physicians recommend colonoscopy consistent with existing guidelines, and whether patients follow through with physicians' recommendations to undergo colonoscopy is a first step. With the often fragmented care of cancer survivors, it may be unclear who is responsible for ensuring adherence to colonoscopy guidelines. Oncologists, primary care providers, and other providers involved in the care of CRC survivors must communicate with each other about their separate responsibilities for detecting second primary cancers. To facilitate communication between providers, it is important to understand whether, as recommended by the recent Institute of Medicine report on cancer survivorship, cancer survivors are provided with a survivorship care plan that includes a recommendation for colonoscopy approximately one year following treatment, whether cancer survivors share this information with their primary care providers, and whether cancer survivors understand the information they receive[36].

## Competing interests

The authors declare that they have no competing interests.

## Authors' contributions

TS, MW, and RS conceived of the study. TS conducted the analysis with assistance from MW and RS. TS, MW, and RS participated in the coordination of the study. TS, JA, NB, CE, JEL, DF, BW, MW, and RS refined the analyses and co-wrote and edited the manuscript. All authors read and approved the final manuscript.

## Pre-publication history

The pre-publication history for this paper can be accessed here:

http://www.biomedcentral.com/1472-6963/10/256/prepub

## References

[B1] Mysliwiec P Cronin K Schatzkin A C. RE Chapter 5: New malignancies following cancer of the colon, rectum, and anus, in New Malignancies Among Cancer Survivors: SEER Cancer Registries 1973-2000 2006 National Cancer Institute: Bethesda, MD 111 144

[B2] Benson AB 2000 update of American Society of Clinical Oncology colorectal cancer surveillance guidelines J Clin Oncol 2000 18 20 3586 8 11032600 10.1200/JCO.2000.18.20.3586

[B3] Simmang CL Practice parameters for detection of colorectal neoplasms. The Standards Committee, The American Society of Colon and Rectal Surgeons Dis Colon Rectum 1999 42 9 1123 9 10.1007/BF02238562 10496550

[B4] Smith RA Cokkinides V Eyre HJ American Cancer Society guidelines for the early detection of cancer, 2003 CA Cancer J Clin 2003 53 1 27 43 10.3322/canjclin.53.1.27 12568442

[B5] Winawer S Colorectal cancer screening and surveillance: clinical guidelines and rationale - update based on new evidence Gastroenterology 2003 124 2 544 60 10.1053/gast.2003.50044 12557158

[B6] The NCCN Colorectal Screening Clinical Practice Guidelines in Oncology (Version 1.2003) 2003 National Comprehensive Cancer Network, Inc 10.6004/jnccn.2003.0009 19764152

[B7] Anthony T Practice parameters for the surveillance and follow-up of patients with colon and rectal cancer Dis Colon Rectum 2004 47 6 807 17 10.1007/s10350-004-0519-x 15108028

[B8] The NCCN Colorectal Screening Clinical Practice Guidelines in Oncology (Version 1.2005) National Comprehensive Cancer Network 2005

[B9] Desch CE Colorectal cancer surveillance: 2005 update of an American Society of Clinical Oncology practice guideline J Clin Oncol 2005 23 33 8512 9 10.1200/JCO.2005.04.0063 16260687

[B10] The NCCN Colorectal Screening Clinical Practice Guidelines in Oncology (Version 1.2006) National Comprehensive Cancer Network 2006

[B11] Rex DK Guidelines for colonoscopy surveillance after cancer resection: a consensus update by the American Cancer Society and the US Multi-Society Task Force on Colorectal Cancer Gastroenterology 2006 130 6 1865 71 10.1053/j.gastro.2006.03.013 16697749

[B12] Elston Lafata J Sociodemographic differences in the receipt of colorectal cancer surveillance care following treatment with curative intent Med Care 2001 39 4 361 72 10.1097/00005650-200104000-00007 11329523

[B13] Ellison GL Racial differences in the receipt of bowel surveillance following potentially curative colorectal cancer surgery Health Serv Res 2003 38 6 Pt 2 1885 903 10.1111/j.1475-6773.2003.00207.x 14727802 PMC1360978

[B14] Rulyak SJ Clinical and sociodemographic factors associated with colon surveillance among patients with a history of colorectal cancer Gastrointest Endosc 2004 59 2 239 47 10.1016/S0016-5107(03)02531-8 14745398

[B15] Elston Lafata J Routine surveillance care after cancer treatment with curative intent Med Care 2005 43 6 592 9 10.1097/01.mlr.0000163656.62562.c4 15908854

[B16] Cooper GS Payes JD Temporal trends in colorectal procedure use after colorectal cancer resection Gastrointest Endosc 2006 64 6 933 40 10.1016/j.gie.2006.08.024 17140901

[B17] Rolnick S Racial and age differences in colon examination surveillance following a diagnosis of colorectal cancer J Natl Cancer Inst Monogr 2005 35 96 101 10.1093/jncimonographs/lgi045 16287893

[B18] Cooper GS Kou TD Reynolds HL Jr Receipt of guideline-recommended follow-up in older colorectal cancer survivors: a Population-based Analysis Cancer 2008 10.1002/cncr.23823 18780338

[B19] Hilsden RJ A retrospective study on the use of post-operative colonoscopy following potentially curative surgery for colorectal cancer in a Canadian province BMC Cancer 2004 4 14 10.1186/1471-2407-4-14 15096279 PMC419354

[B20] Cooper GSM Geographic and patient variation among Medicare beneficiaries in the use of follow-up testing after surgery for nonmetastatic colorectal carcinoma Cancer 1999 85 10 2124 31 10.1002/(SICI)1097-0142(19990515)85:10<2124::AID-CNCR5>3.0.CO;2-L 10326689

[B21] Knopf KB Bowel surveillance patterns after a diagnosis of colorectal cancer in Medicare beneficiaries Gastrointest Endosc 2001 54 5 563 71 10.1067/mge.2001.118949 11677471

[B22] Cancer Care Outcomes Research & Surveillance Consortium 2007 http://healthservices.cancer.gov/cancors/April 9, 2008

[B23] Ayanian JZ Understanding Cancer Treatment and Outcomes: The Cancer Care Outcomes Research and Surveillance Consortium J Clin Oncol 2004 22 15 2992 2996 10.1200/JCO.2004.06.020 15284250

[B24] Wagner EH Building a research consortium of large health systems: the Cancer Research Network J Natl Cancer Inst Monogr 2005 35 3 11 10.1093/jncimonographs/lgi032 16287880

[B25] The NCCN Colorectal Screening Clinical Practice Guidelines in Oncology (Version 1.2004) National Comprehensive Cancer Network 2004

[B26] Johnston A Validation of a comorbidity education program J of Registry Management 2001 28 3 125 131

[B27] Piccirillo J Importance of comorbidity in head and neck cancer Laryngoscope 2000 110 4 593 602 10.1097/00005537-200004000-00011 10764003

[B28] Piccirillo J The measurement of comorbidity by cancer registries J of Registry Management 2003 30 4 8 14

[B29] Stata Statistical Software: Release 10 StataCorp LP 2007 College Station, TX

[B30] Hosmer DW Lemeshow S Applied Logistic Regression 2000 2 New York: Wiley-Interscience

[B31] Madden D Nolan A Nolan B GP reimbursement and visiting behaviour in Ireland Health Econ 2005 14 10 1047 60 10.1002/hec.995 15791674

[B32] Davila RE ASGE guideline: colorectal cancer screening and surveillance Gastrointest Endosc 2006 63 4 546 57 10.1016/j.gie.2006.02.002 16564851

[B33] Cooper GS Koroukian SM Geographic variation among Medicare beneficiaries in the use of colorectal carcinoma screening procedures Am J Gastroenterol 2004 99 8 1544 50 10.1111/j.1572-0241.2004.30902.x 15307875

[B34] Earle CC Neville BA Under use of necessary care among cancer survivors Cancer 2004 101 8 1712 9 10.1002/cncr.20560 15386307

[B35] Snyder CF Preventive care for colorectal cancer survivors: a 5-year longitudinal study J Clin Oncol 2008 26 7 1073 9 10.1200/JCO.2007.11.9859 18309941

[B36] Hewitt M, Greenfield S, Stovall E From Cancer Patient to Cancer Survivor: Lost in Transition 2006 Institute of Medicine and National Research Council

[B37] Cooper GS Use of guideline recommended follow-up care in cancer survivors: routine or diagnostic indications? Med Care 2006 44 6 590 4 10.1097/01.mlr.0000215902.50543.77 16708008

